# Knee Taping and the Countermovement Jump: Implications for Reactive Strength, Power, and Jump Mechanics

**DOI:** 10.3390/jfmk10040418

**Published:** 2025-10-23

**Authors:** Kendra Taryn Szeles, Andrew Green

**Affiliations:** Department of Sport and Movement Studies, Faculty of Health Science, University of Johannesburg, Johannesburg 2028, Gauteng, South Africa; kendraszeles@gmail.com

**Keywords:** knee strapping, Statistical Parametric Mapping, jump performance, Kinesio Tape, Biomechanics

## Abstract

**Background**: The use of knee taping is widely used to enhance stability and landing performance. However, its impact on jumping performance, a key sports performance determinant, remains unknown. **Objective**: This study aimed to evaluate the effects of knee taping on continuous biomechanics during the countermovement jump (CMJ). **Methods**: Nineteen recreational female netball players (age 22 ± 2.69 years; height 167.76 ± 7.47 cm; mass 63.32 ± 10.57 kg) performed CMJs under four taping conditions—no tape (NT), rigid tape (RT), dynamic tape (DT), and kinesio tape (KT). All participants completed all four conditions. Continuous biomechanical data were analysed using one-dimensional statistical parametric mapping (SPM1d) with repeated measures ANOVA (*p* < 0.05). **Results**: KT significantly reduced time to take-off (from 2.01 ± 0.67 s with NT to 1.26 ± 0.61 s with KT, *p* < 0.001) and increased modified reactive strength index (from 0.12 ± 0.05 with NT to 0.21 ± 0.06 with KT, *p* < 0.001). RT increased braking phase velocity compared to NT (−1.53 ± 0.57 m/s vs. −1.69 ± 0.62 m/s, *p* = 0.01). SPM1d revealed significant kinematic changes across conditions, including reduced ankle flexion and hip flexion with KT, and increased knee rotation with RT and DT. **Conclusions**: Knee taping modified joint kinematics without enhancing maximal outputs such as power or jump height. KT improved reactive strength indices, suggesting potential benefits for rapid jump performance, while RT and DT mainly altered joint coordination.

## 1. Introduction

Explosive lower-limb power forms the basis for performance in many sports, particularly netball which requires frequent jumping, sprinting, and rapid changes of direction (1). Explosiveness is the ability to generate and apply force efficiently through the stretch–shortening cycle (SSC) and contributes not only to jump height but also to the speed of transitions between offensive and defensive actions, which often determine competitive outcomes [1,2]. Accordingly, reliable measures of power-related performance are essential for evaluating athletes’ neuromuscular function and monitoring adaptations to interventions.

The countermovement jump (CMJ) is one of the most widely applied assessments of explosive lower-limb performance and SSC efficiency [3,4]. Although it does not provide a direct measure of muscular power, the CMJ yields performance indices such as jump height, time to take-off (TTO), and the modified reactive strength index (RSImod). These indices act as proxies for neuromuscular efficiency and power transfer, with RSImod in particular distinguishing between higher- and lower-performing athletes by reflecting their capacity to rapidly transition from eccentric to concentric muscle actions [5,6,7].

The CMJ consists of sequential unweighting, braking, and propulsive phases before take-off [8,9,10]. Traditional analyses have focused on discrete variables, such as peak ground reaction force (GRF) or maximum velocity [3,4]. While these measures are informative, they capture only isolated time points and may overlook adaptations that emerge across the entire movement cycle. Statistical parametric mapping (SPM) offers a solution by enabling the continuous analysis of normalised force and kinematic time, providing greater sensitivity to subtle alterations in joint motion and force application that discrete approaches may miss [11,12,13]. This approach is particularly valuable when evaluating interventions that may affect coordination across phases of movement, such as joint taping.

Athletes frequently use taping strategies to support joint function, both in injury management and in performance contexts. The three most common techniques are rigid tape (RT), dynamic tape (DT), and kinesio tape (KT), which differ in their mechanisms of action. RT is designed to mechanically restrict excessive joint motion and improve stability [14]. DT provides strong elastic resistance and recoil, supporting controlled movement and load absorption [15]. KT, by contrast, is thought to act neurophysiologically, enhancing proprioceptive input and joint position sense [16]. In rehabilitation, RT and KT have been shown to reduce pain, improve patellofemoral alignment, and enhance proprioception in injured populations [14,16]. DT has also demonstrated potential benefits for load absorption and support during return-to-play protocols [15]. Although these methods are widely applied, evidence for their effects on jump performance remains inconsistent. Some studies suggest KT may improve proprioception and vertical jump outcomes [17,18], while others report no measurable performance benefits [19,20]. Direct comparisons between RT, DT, and KT in the context of the CMJ are limited, leaving a knowledge gap regarding their capacity to influence explosive performance in uninjured athletes.

It was hypothesised that KT would improve reactive strength performance through enhanced proprioceptive feedback, whereas RT and DT would primarily modify lower-limb kinematics. The objective of this study was to evaluate the acute effects of RT, DT, and KT on CMJ performance, focusing on discrete measures of power-related outcomes alongside continuous biomechanical analysis of lower-limb joint kinematics and kinetics using SPM.

## 2. Materials and Methods

### 2.1. Participants

This was a repeated-measures, within-subject experimental study conducted in the Biomechanics Laboratory at the University of Johannesburg.

An a priori power calculation was conducted using G*Power software version 3.1.9.7, based on a large effect size (f = 0.4; η^2^ ≈ 0.5) with α = 0.05 and a power of 0.95, indicating a minimum of 15 participants required.

Consequently, 19 injury-free female netball players (age 22 ± 2.69 years, height: 167.76 ± 7.47 cm, mass: 63.32 ± 10.57 kg) participated in this investigation. Height was measured using a wall-mounted stadiometer (Seca 213, Hamburg, Germany), and body mass was recorded using a calibrated digital scale (Seca Robusta 813, Hamburg, Germany). Age and right leg dominance were self-reported. Taping was applied to the dominant limb in every case. Participants signed an informed consent form, and institutional ethics approval was granted by the Faculty of Health Sciences Research Ethics Committee at the University of Johannesburg prior to the commencement of the study (REC-1760-2022). This was not a registered clinical trial.

### 2.2. Procedure

**Figure 1:**
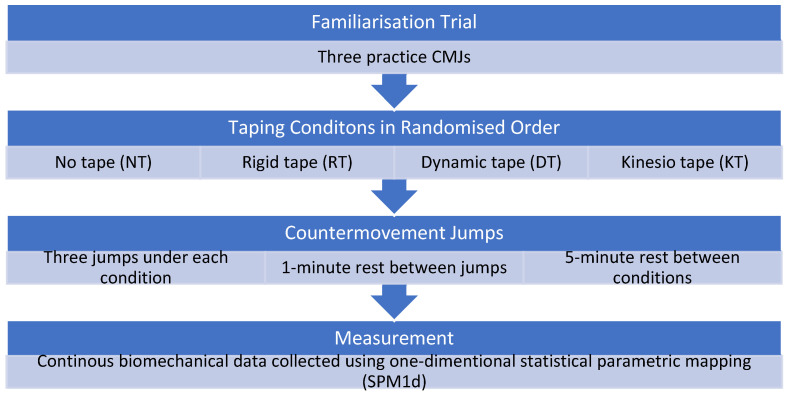
Overview of the study protocol. All 19 participants completed a familiarisation trial, followed by three countermovement jumps under each of the four taping conditions (NT, RT, DT, KT) in a randomised order.

Participants were required to perform three countermovement jumps (CMJ) under four knee-taping conditions: no tape (NT), rigid tape (RT), dynamic tape (DT), and kinesio tape (KT) (Figure 1). All participants completed all four conditions. Participants were familiarised with the CMJ protocol by completing three practice jumps immediately prior to testing. The purpose of the familiarisation trials was to ensure participants were able to reach the desired squat depth and execute explosive jumps. The order of the four taping conditions (NT, RT, DT, KT) was randomised across participants to reduce potential order effects. Each jump was separated by a one-minute rest period, and a five-minute interval was provided between conditions to minimise fatigue effects. Jumps were performed with hands placed on hips and a self-selected countermovement depth to optimise take-off height. Participants were instructed to perform the downward and upward phases rapidly and continuously to maximise jump performance [3].

### 2.3. Taping Application

**Figure 2:**
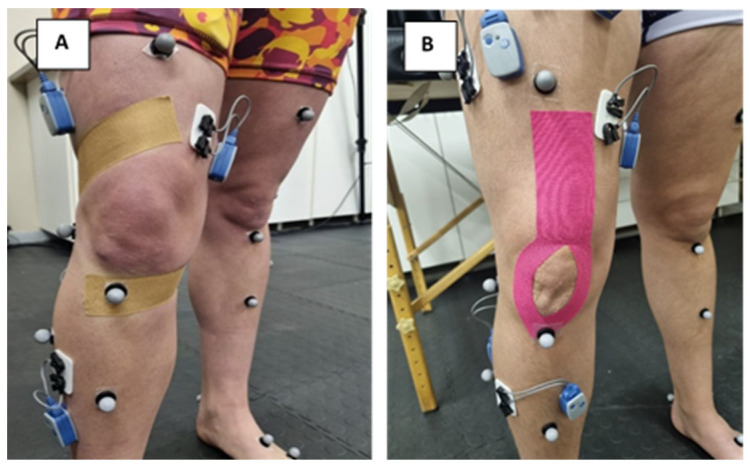
Taping techniques—(**A**) Mulligan’s Taping Technique (**B**) “Y” Formation Technique.

Three taping conditions were applied, each selected for its distinct biomechanical or neurosensory function (Figure 2). RT was used to provide mechanical restrictions of excessive knee movement, while DT was selected to provide elastic recoil and load absorption. RT and DT were applied using Mulligan’s taping technique [20,21]. For this technique, participants were positioned with the hip slightly internally rotated, and the knee flexed at 25°. Tape was applied from the fibular head across the tibia with an internal torsion force and anchored posterior to the medial knee joint line before being fixed over the posterior aspect. Mulligan’s taping technique is intended to limit undesirable tibial rotation and anterior translation.

KT (Kinesio^®^) was used to enhance proprioceptive feedback and neuromuscular control. KT was applied using the “Y” formation technique [16]. For this technique, participants were positioned with the quadriceps under stretch. Tape was anchored from the anterior inferior iliac spine to the tibial tuberosity, with 50–75% stretch applied to the mid-section and no stretch at the extremities. The “Y” formation technique is intended to improve sensorimotor awareness during movement.

All taping was applied by the same practitioner to ensure consistency, and testing commenced within five minutes of tape application.

### 2.4. Biomechanical Analysis

All kinematics were recorded at 200 Hz using a 10-camera motion capture system (Vicon Vero 2.2, Vicon Motion Systems Ltd., Oxfordshire, UK) with the CGM 2.5 model markerset. Simultaneous ground reaction forces were recorded at 1000 Hz using two multi-axis force platforms (Bertec, Columbus, OH, USA). Sub-millimetre kinematic calibration, tracking, and synchronisation of kinetics were performed in Nexus software (Vicon Vero 2.2 (Vicon Motion Systems Ltd., UK)).

### 2.5. Data Analysis

The CMJ profile, from initiation to toe-off, was divided into three phases: (1) the unweighting phase, from movement initiation until minimum vertical ground reaction force (vGRF) was reached; (2) the braking phase, from minimum vGRF until CoM velocity reached zero, representing deceleration; and (3) the propulsion phase, from the lowest CoM position to toe-off, corresponding with positive CoM velocity [6,8]. These definitions are consistent with previous descriptions of CMJ mechanics and phase transitions [9,10].

Kinetic and kinematic values of the three jump attempts were time-corrected, and the best of three jumps, being the one with the greatest power output, was selected for data analysis. Jump height was calculated using the momentum impulse method via the second integral of net acceleration [4]. TTO was defined as the duration from initiation of the downward movement to toe-off [8]. RSImod was used to determine the interaction between jump height and TTO, calculated as jump height divided by TTO [5,7]. Full waveform analyses of CMJ trials were conducted by normalising each participant’s kinetic-time curves (force, velocity, and displacement) to 101 nodes (0–100% of movement time).

### 2.6. Statistical Analysis

All discrete CMJ metrics (maximum take-off force, take-off velocity, take-off momentum, braking phase velocity, peak propulsive power, jump height, TTO, and RSImod) were first evaluated for normality using the Shapiro–Wilk test. Variables meeting assumptions of normality are presented as mean ± standard deviation, whereas non-normally distributed variables are presented as median and interquartile range.

Normally distributed variables were analysed using repeated measures ANOVA to compare within-participant differences across the four taping conditions. Where significant main effects were detected, pairwise comparisons were conducted using Bonferroni-adjusted post hoc tests, and effect sizes were reported as eta-squared (η^2^).

Non-normally distributed variables were compared using Friedman’s test with Wilcoxon signed-rank tests for post hoc comparisons. Effect sizes were expressed as Kendall’s W, with thresholds of 0.1–<0.3 = small, 0.3–0.5 = moderate, and ≥0.5 = large effects.

Continuous biomechanical data were analysed using non-parametric SPM1d repeated measures ANOVA (*p* < 0.05) in MATLAB (Pataky, 2022). Post hoc analysis was conducted in the form of non-parametric paired t-tests using a Bonferroni correction (*p* < 0.0085).

## 3. Results

**Figure 3:**
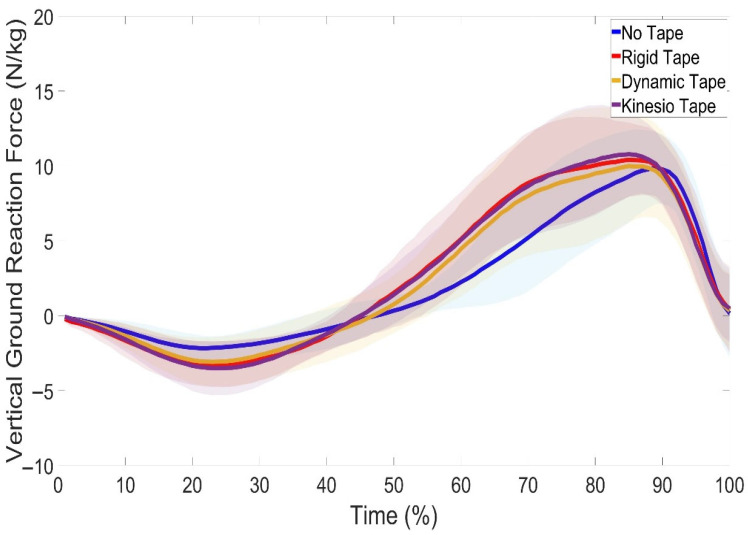
Vertical ground reaction force (GRF) during the countermovement jump under no tape (NT), rigid tape (RT), dynamic tape (DT), and kinesio tape (KT) conditions in nineteen female recreational netball players. Shaded regions indicate the variability around the mean/median. Dotted lines indicate phases where significant differences were detected by SPM1d (*p* < 0.05). No significant differences in GRF were observed across conditions.

The CMJ kinetics revealed no significant changes in GRF across all conditions (Figure 3). No significant changes were found in maximum take-off force (*p* = 0.919, η^2^ = 0.006), take-off velocity (*p* = 0.592, η^2^ = 0.025), take-off momentum (*p* = 0.826, η^2^ = 0.012), peak propulsive power (*p* = 0.50, η^2^ = 0.030), or jump height (*p* = 0.284, η^2^ = 0.054). The effect sizes for these outcomes were small, suggesting that the absence of statistical differences was accompanied by little or no practical impact across conditions.

Significant changes were seen in TTO (*p* < 0.001, η^2^ = 0.255) (Table 1), with a moderate effect, where KT resulted in the quickest TTO compared to NT (*p* < 0.001), DT (*p* = 0.014), and RT (*p* = 0.002). Similarly, RSImod showed significant differences (*p* < 0.001, W = 0.516) with a large effect size, with KT reporting the highest RSImod values compared to NT (*p* < 0.001) and RT (*p* = 0.003). Further, significant changes were observed in braking phase velocity (*p* = 0.01, W = 0.198), where RT reported faster braking phase velocity compared to NT (*p* = 0.01). Although the effect size was small, this finding represents a statistically significant change, indicating a small-magnitude but potentially meaningful adjustment in braking performance.

**Figure 4:**
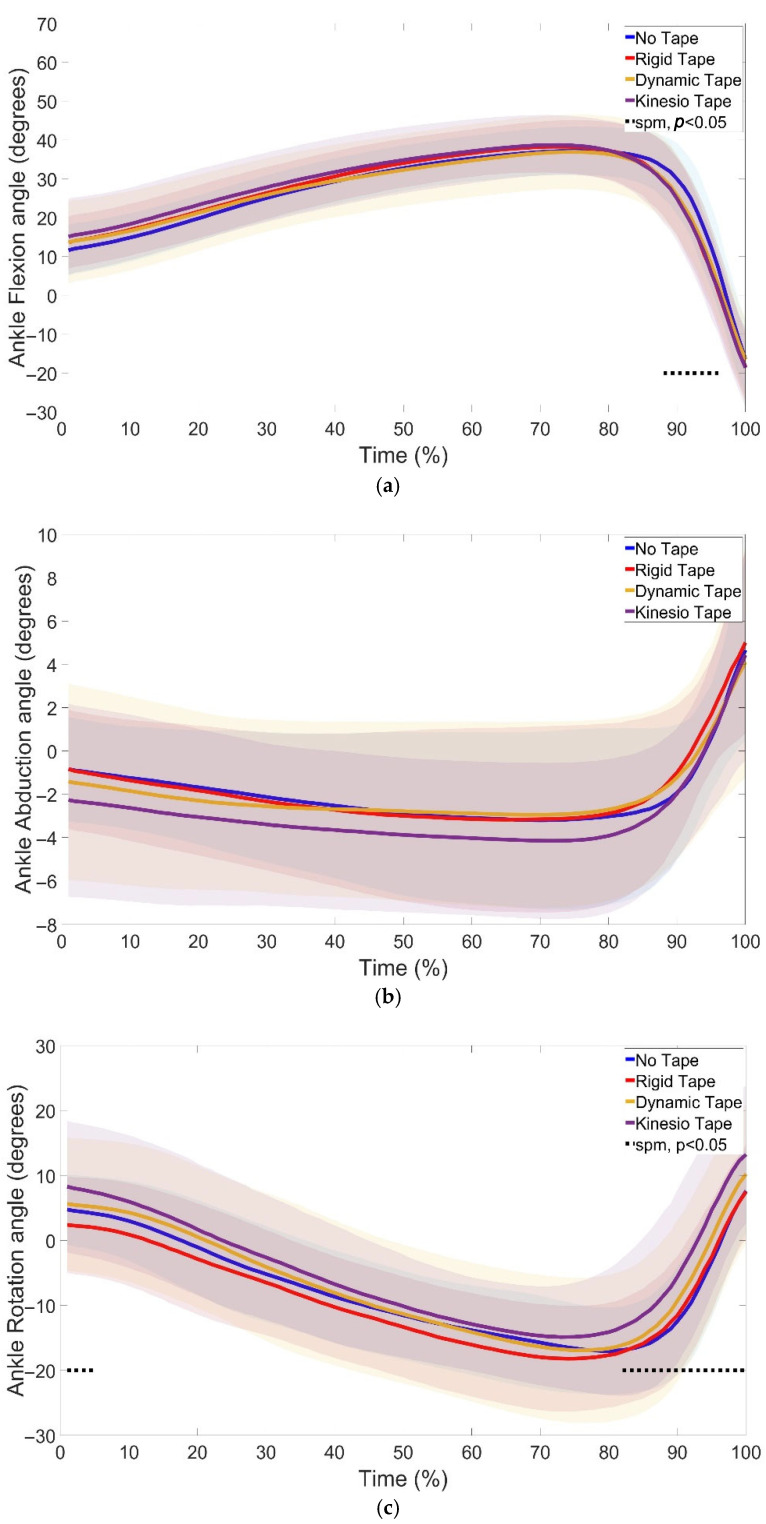
Ankle flexion (**a**), abduction (**b**), and rotation (**c**) during the countermovement jump under no tape (NT), rigid tape (RT), dynamic tape (DT), and kinesio tape (KT) conditions. Shaded regions indicate the variability around the mean/median. Dotted lines indicate phases where significant differences were detected by SPM1d (*p* < 0.05). KT decreased ankle flexion compared to NT (88–96%) and increased ankle rotation compared to NT (86–97%) and RT (1–5%, 82–100%).

**Figure 5:**
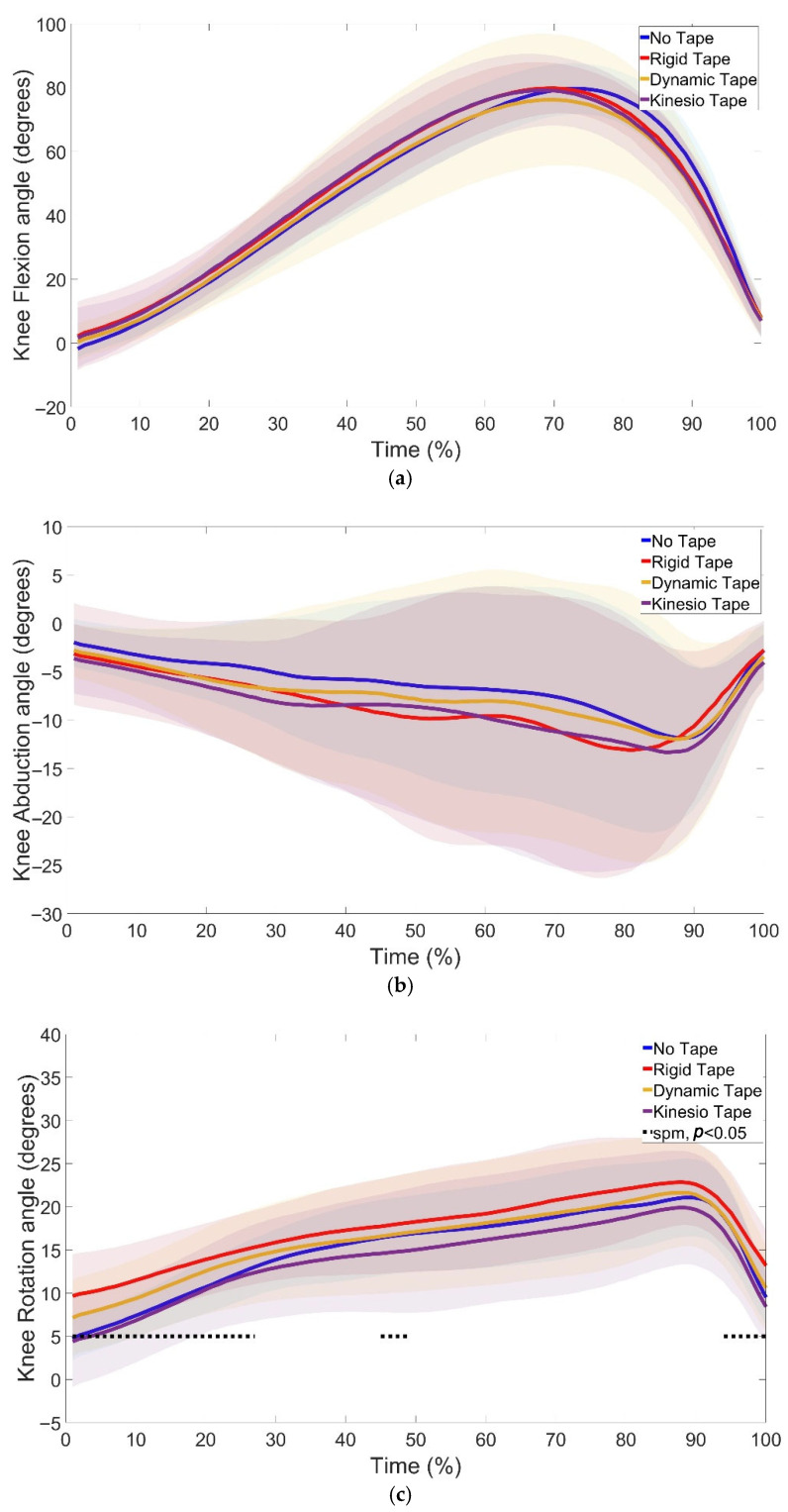
Knee flexion (**a**), abduction (**b**), and rotation (**c**) during the countermovement jump under NT, RT, DT, and KT conditions. Shaded regions indicate the variability around the mean/median. Dotted lines indicate phases where significant differences were detected by SPM1d (*p* < 0.05). Significant differences in knee rotation were found between NT and RT (1–26%, 97–100%), RT and DT (1–11%, 98–100%), RT and KT (1–26%, 43–100%), and DT and KT (1–21%, 97–100%).

**Figure 6:**
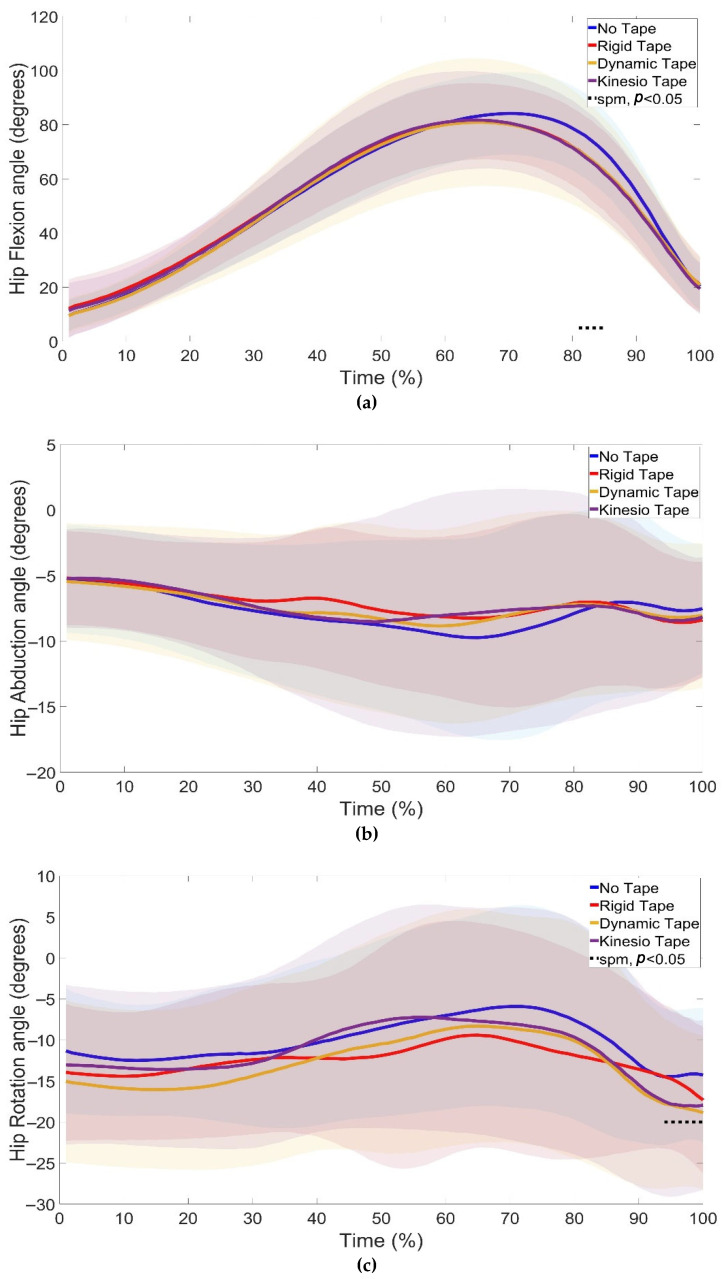
Hip flexion (**a**), abduction (**b**), and rotation (**c**) during the countermovement jump under NT, RT, DT, and KT conditions. Shaded regions indicate the variability around the mean/median. Dotted lines indicate phases where significant differences were detected by SPM1d (*p* < 0.05). KT differed from NT in hip flexion (81–85%), while hip rotation differed between NT and RT (99–100%), NT and DT (94–100%), and NT and KT (94–100%).

SPM1d analyses of joint kinematics revealed significant changes in ankle (Figure 4), knee (Figure 5), and hip (Figure 6) angles under different taping conditions.

At the ankle, no significant differences were seen in ankle abduction (Figure 4b). KT significantly decreased flexion compared to NT (88–96%, *p* = 0.01) (Figure 4a) and increased rotation compared to NT (phases 86–97%) and RT (phases 1–5%; 82–100%) (Figure 4c). The changes seen at the ankle with KT were accompanied by a reduction in TTO (Table 1).

At the knee, no significant changes were found in knee flexion (Figure 5a) and abduction (Figure 5b). Significant differences in rotation were found (1–27%, *p* = 0.010; 45–49%, *p* = 0.04; 94–100%, *p* = 0.010) between NT vs. RT (phases 1–26%; 97–100%), RT vs. DT (phases 1–11%; 98–100%), RT vs. KT (phases 1–26%; 43–100%), and DT vs. KT (phases 1–21%; 97–100%) (Figure 5c). The increased rotation at the knee co-occurred with higher RSImod in RT, DT, and KT, and faster braking phase velocity with RT (Table 1).

At the hip, no significant differences were seen in hip abduction (Figure 6b). Significant changes in flexion (81–85%, *p* = 0.04) were observed between NT and KT (phases 81–85%) (Figure 6a) and rotation (94–100%, *p* = 0.04) between NT vs. RT (phases 99 = 100%), NT vs. DT (phases 94–100%), and NT vs. KT (phases 94–100%) (Figure 6c). The late phase hip rotation differences aligned with faster braking phase velocity (Table 1).

## 4. Discussion

The purpose of this study was to investigate the effects of different knee taping conditions on lower-limb performance and joint kinematics during the CMJ. Significant changes were found in TTO, RSImod, and braking phase velocity. In addition, SPM1d analyses revealed significant kinematic differences at the ankle, knee, and hip across the movement cycle. No significant differences were observed in GRF (Figure 3), jump height, max take-off force, or propulsive power (Table 1). Further, the taping conditions did not improve or impede take-off velocity or take-off momentum performance.

Previous studies have suggested that KT may influence performance-related indices by facilitating quicker SSC transitions [17,18]. Higher RSImod is typically linked to higher-performing jumpers who demonstrate shorter TTO and greater braking force development [7]. Consistent with this literature, the present study found that KT produced notable improvements in TTO and RSImod compared to NT and RT with a moderate to large effect size. These findings suggest that KT may enhance proprioceptive input and motor control, thereby enabling more efficient SSC utilisation. However, as in previous reports [19], no significant improvements were observed in jump height and propulsive power, indicating that KT primarily influences rapid reactive strategies rather than maximal power output.

RT has been shown to mechanically restrict knee joint motion and improve stability [14]. Such a restriction may influence CMJ mechanics by altering descent strategies. In the current study, RT was associated with faster downward velocity during the braking phase compared to NT. Although this adaptation aligns with its intended role in joint control, the small effect size and lack of change in propulsive outcomes indicate limited performance benefit. This pattern suggests that RT altered coordination without enhancing explosive output, which is consistent with earlier findings that Mulligan’s taping modifies knee rotation without measurable improvements in jump performance [20].

Previous research has highlighted the value of continuous analyses in detecting subtle alterations in joint motion [13]. In line with this, the present study identified significant kinematic changes at the ankle (Figure 4), knee (Figure 5), and hip (Figure 6) across the CMJ cycle.

At the ankle, KT reduced flexion and increased rotation compared to NT and RT, particularly during the propulsive phase. These ankle kinematic changes occurred alongside the shorter TTO observed with KT. Whether these adaptations directly influence SSC transitions remains uncertain and warrants further investigation, but KT has been suggested to enhance proprioceptive input and movement efficiency [22,23].

At the knee, RT and DT increased joint rotation throughout the unweighting and propulsion phases, aligning with reports by [24] that Mulligan’s taping increases knee rotation and alters joint alignment during dynamic movements. While these kinematic adaptations reflect altered coordination, they were not linked to performance improvements.

At the hip, KT reduced flexion in the propulsive phase, while all three taping conditions increased hip rotation relative to NT in the final stages of the jump. These adaptations suggest that changes initiated at the knee influenced movement across the kinetic chain, reinforcing the interdependence of ankle, knee, and hip coordination [25]. Although these modifications altered joint mechanics, they were not accompanied by increases in jump height or propulsive power, indicating that the influence of taping on coordination does not necessarily translate into enhanced explosive jumping performance.

These findings indicate that knee taping modifies lower limb kinematics during a CMJ without improving maximal power outputs. KT reduced TTO and increased RSImod, supporting more efficient SSC utilisation, whereas RT and DT primarily influenced kinematic patterns without producing measurable performance benefits.

## 5. Implications

This study highlights that knee taping influences not only the taped joint but also coordination across the ankle and hip, reinforcing the importance of evaluating lower-limb movement strategies holistically. While RT and DT modified joint kinematics, these changes were not associated with improvements in performance metrics such as jump height or propulsive power. By contrast, KT significantly improved TTO and RSImod, indicating potential benefits for reactive strength and SSC efficiency. Importantly, none of the taping conditions reduced performance, suggesting that knee taping can be used as an injury prevention or return-to-play strategy without compromising explosive jump performance.

From a practical perspective, these findings suggest that KT may be useful in contexts where rapid SSC transitions and reactive performance are prioritised, such as in netball, where repeated jumping or quick takeoffs are required. In addition, the observed alterations in joint ROM with RT, DT, and KT may support injury prevention protocols by placing the knee in more favourable positions for load absorption and control of excessive joint motion. Although taping should not be considered a direct performance-enhancing intervention for explosive power, its value may lie in supporting athletes in refining their movement strategies and reactive capabilities without compromising overall performance.

## 6. Conclusions

Knee taping influenced movement strategies during the CMJ without enhancing maximal power production. Importantly, none of the taping conditions reduced performance, indicating that knee taping did not impose kinetic drawbacks. KT demonstrated potential benefits for reactive strength and rapid SSC transitions, while RT and DT primarily influenced joint coordination. These findings confirm that knee taping modifies movement strategy rather than increasing kinetic outputs such as force, power, or jump height.

## 7. Limitations

CMJ performance in a controlled setting does not account for fatigue effects or dynamic in-game movements that could alter taping efficacy. Future studies could investigate how taping influences movement patterns under fatigue or repeated jumps over time. Additionally, the selected sample was not injured or returning from injury. Future studies could investigate the effects of taping on a sample returning from injury. Participants were not blinded to the taping condition, meaning that placebo or expectation effects cannot be ruled out. Finally, the sample consisted of healthy, recreational female netball players, which limits the generalisability of these findings to male athletes. Future studies could investigate the difference in the effects between female and male athletes.

## Figures and Tables

**Table 1:** Discrete countermovement jump performance indices of 19 female netball players under four taping conditions of No Tape (NT), Rigid Tape (RT), Dynamic Tape (DT), and Kinesio Tape (KT).

	NT	RT	DT	KT
Maximum take-off force (N/kg)	13.40 ± 2.63	13.31 ± 2.73	13.19 ± 2.79	13.79 ± 3.33
Take-off velocity (m/s)	1.85 ± 0.22	1.94 ± 0.19	1.95 ± 0.19	1.93 ± 0.23
Take-off momentum (kg.m/s)	115.96 ± 17.46	121.69 ± 20.75	122.14 ± 21.92	121.53 ± 24.64
Time to take-off (s)	2.19 (1.79 to 2.29) †	2.02 (1.72 to 2.41) †	1.97 (1.44 to 2.25) †	1.49 (1.29 to 1.59)
Peak propulsive power (W)	868.95 ± 237.96	939.89 ± 251.51	942.36 ± 248.60	839.70 ± 259.10
Breaking phase velocity (m/s)	−1.72 (−1.91 to −1.36)	−1.88 (−2.04 to −1.58) *	−1.81 (−1.99 to −0.74)	−1.68 (−2.04 to −0.66)
Jump height (m)	0.26 ± 0.04	0.28 ± 0.04	0.27 ± 0.03	0.28 ± 0.04
Modified reactive strength index	0.12 (0.10 to 0.15)†	0.14 (0.11 to 0.17)†	0.15 (0.13 to 0.17)	0.21 (0.17 to 0.22)

Data are presented as mean ± SD for normally distributed variables and as median (IQR) for non-normally distributed variables. † Significant from KT. * Significant from NT.

## Data Availability

The original contributions presented in this study are included in the article. Data are available upon reasonable request from the corresponding author.

## Abbreviations

The following abbreviations are used in this manuscript:

CMJ	Countermovement jump
CoM	Centre of mass
DT	Dynamic tape
GRF	Ground reaction force
KT	Kinesio tape
NT	No tape
RT	Rigid tape
RSImod	Modified reactive strength index
SCC	Stretch–shortening cycle
SPM	Statistical parametric mapping
SPM1d	One-dimensional statistical parametric mapping
TTO	Time to takeoff
vGRF	Vertical ground reaction force
